# Splenic Marginal Zone Lymphoma in a Patient With Antiphospholipid Syndrome Treated With Rituximab: A Case Report

**DOI:** 10.7759/cureus.55374

**Published:** 2024-03-02

**Authors:** Laxman Wagle, Dhiraj R Regmi, Samaj Adhikari, Parmartha Basnyat, Abhishek Kalla

**Affiliations:** 1 Internal Medicine, Ascension Saint Agnes Hospital, Baltimore, USA; 2 Internal Medicine, Tanahun Sewa Hospital, Tanahun, NPL; 3 Internal Medicine, Interfaith Medical Center, Brooklyn, USA; 4 Hematology and Oncology, Ascension Saint Agnes Hospital, Baltimore, USA

**Keywords:** non-hodgkin’s lymphomas, antiphospholipid antibody (apla), rituximab therapy, splenic marginal zone lymphoma, antiphospholipid antibody syndrome (aps)

## Abstract

Splenic marginal zone lymphoma (SMZL) usually presents with splenomegaly or symptoms related to cytopenia. We report a case of a 56-year-old female with previously diagnosed antiphospholipid syndrome (APS) on warfarin therapy who initially presented with abdominal pain and was found to have massive splenomegaly and splenic infarction on CT imaging. Initial clinical presentations and imaging findings were attributed to the subtherapeutic coagulation profile. The patient was later diagnosed with SMZL following workup for pancytopenia including bone marrow biopsy, flow cytometry, and PET scan. Cytopenias, splenomegaly, and abnormal metabolic activity in the spleen on the PET scan improved after treatment with four cycles of weekly rituximab. Our report presents a case of a patient with longstanding APS presenting with splenic infarction and pancytopenia who was subsequently diagnosed with SMZL and successfully treated with rituximab.

## Introduction

Splenic marginal zone lymphoma (SMZL) is a rare subtype of non-Hodgkin's lymphoma (NHL) that originates from B memory lymphocytes that are present in the marginal zone of secondary lymphoid follicles [1]. A population-based study in the United States (2001-2008) showed the overall annual age-adjusted incidence of SMZL to be 0.13 per 10,000 persons per year comprising 0.6% percent of cases of NHL [2]. Typically, patients with SMZL have enlarged spleen, lymphocytosis, and cytopenias. Analysis of pathological cells in bone marrow is sufficient for the diagnosis of SMZL rather than blood or spleen analysis [1]. Bone marrow infiltration is a very common finding (83-100%), although circulating cells are detected much less frequently (29-75%) [3]. A high incidence of antiphospholipid antibodies has been reported in newly diagnosed patients with lymphoma. In a study by Kungwankiattichai et. al., more than one-third of patients were found to have antiphospholipid antibodies (APLAs) [4]. There have been limited reports of antiphospholipid syndrome (APS) among patients with SMZL [5-7]. Rituximab, chemotherapy, and splenectomy are the main modalities of treatment for SMZL [8]. 

We report a case of a 56-year-old female with a history of APS and ischemic stroke whose initial diagnosis was thought to be splenic infarction due to the subtherapeutic coagulation profile. However, her pancytopenia continued to worsen on follow-up and further histopathological analysis of bone marrow biopsy, peripheral blood smear, and PET scan revealed the diagnosis of SMZL.

## Case presentation

A 56-year-old African American woman with a history of hyperlipidemia, APS, cerebrovascular accident (diagnosed at the age of 26) with residual expressive aphasia and weakness in the right leg, and hypothyroidism came to the hemato-oncology clinic after a recent hospitalization for massive splenomegaly (16.9 cm) and multiple splenic infarcts which were revealed in CT imaging. 

She was taking levetiracetam extended release tablet 1000 mg once a day, warfarin 5 mg once a day, atorvastatin 10 mg once a day, and levothyroxine 75 micrograms once a day. She is allergic to trimethoprim/sulfamethoxazole and penicillin, both of which lead to hives. Her family history was positive for hypertension in her mother; however, she did not report any family history of cancer or autoimmune diseases. She denied smoking cigarettes and drug or alcohol use.

Five months ago, she presented to the emergency room with a history of progressive right upper quadrant abdominal pain of one-month duration. Pain was non-radiating with no relation to food intake and respiration. Her abdominal pain was associated with a decrease in appetite and mild nausea. She denied diarrhea, melena, and blood in the stool or recent trauma to the abdomen. She denied weight loss, night sweats, fatigue, or fever. In the emergency room, her vitals recorded were a temperature of 98.7 Fahrenheit, a heart rate of 106 beats per minute and regular, a respiratory rate of 20 breaths per minute, a blood pressure of 106/76 mm of Hg, and a body mass index of 38.5. Initial laboratory results are demonstrated in Table 1, which was remarkable for bicytopenia.

**Table 1:** Follow-up basic laboratory reports in the six months after the initial presentation Units of WBC: thousand/microliter (K/µL), hemoglobin: gram/deciliter (g/dl), and platelet: thousand/microliter (K/µL).

	Initial visit	After five months	After blood transfusion and Iron sucrose infusion (twice)
White blood cells (K/µL)	4	1.7	1.5
Hemoglobin (g/dl)	8.5	6 .8	7.8
Platelets (K/µL)	101	73	77

During the hospitalization, a computed tomography scan (CT scan) of the abdomen with contrast showed splenomegaly (size: 16.9 cm) with multiple splenic infarcts which were initially thought secondary to subtherapeutic anticoagulation profile in a patient with underlying antiphospholipid syndrome. The patient's INR level was subtherapeutic during the previous one to two months before she presented to the emergency room and was in the range of 1.3-1.7. 

The patient's clinical presentation and imaging findings were thought to be secondary to the subtherapeutic antitherapeutic coagulation profile, and the patient was discharged on warfarin bridging with an enoxaparin therapeutic dose and advised to follow in a hemato-oncology clinic on an outpatient basis.

On the patient’s first follow-up visit with the hemato-oncology clinic, her vital signs were temperature was 98.9 Fahrenheit, heart rate 109 beats per minute and regular, respiratory rate 18 breaths per minute, blood pressure 114/80 mm of hg, and body mass index of 37.79. The patient was alert, awake, and oriented to time, place, and person. Mild conjunctival pallor was noted without any scleral icterus. Lungs were clear and normal; s1 and s2 were heard with no murmur on cardiovascular examination. Splenomegaly was noted up to the umbilicus, and bowel sounds were present. No cervical, inguinal, or axillary lymphadenopathy was detected. The patient’s follow-up basic laboratory work within the six months of her hospitalization is shown in Table 1. The patient developed worsening pancytopenia on follow-up visits.

On the first follow-up visit, the complete metabolic profile was unremarkable. Liver function tests were normal. The iron profile on the fifth-month follow-up visit showed low serum iron, low total iron binding capacity, low transferrin saturation, and elevated ferritin. Lab work is shown in Table 2. The patient was transfused with one unit of packed red blood cells and two doses of weekly 300 mg of iron sucrose which improved the patient's hemoglobin but still had pancytopenia. Serum protein electrophoresis showed increased alpha-1 region, immunofluorescent gel showed a normal pattern with no monoclonal proteins, free kappa light chain, quantitative 68.04 mg/L, free lambda light chain, quantitative 13.31 mg/L, with a free kappa/lambda ratio of 5.11.

**Table 2:** Other follow-up lab values at five months along with their reference range mEq/L: milliequivalents per liter; mg/dl: milligrams per deciliter; µ/dl: micrograms per deciliter; ng/dl: nanograms per deciliter; pg/ml: picograms per milliliter; ng/ml: nanograms per milliliter

Labs	Values	Normal Range
Sodium	141 mEq/L	135-145 mEq/L
Potassium	4.2 mEq/L	3.5-5 mEq/L
Blood Urea Nitrogen	12 mg/dl	8-21 mg/dl
Creatinine	0.9 mg/dl	0.6-1.2 mg/dl
Iron	30 µ/dl	60-170 µ/dl
Total Iron Binding Capacity	234 µ/dl	240-450 µ/dl
Transferrin	13 %	20-50 %
Ferritin	193 ng/dl	12-150 ng/dl
Vitamin B12	425 pg/ml	160-950 pg/ml
Folate	>20ng/ml	2.5-20 ng/ml
Immunoglobulin G (IgG)	771 mg/dl	700-1600 mg/dl
Immunoglobulin A (IgA)	70 mg/dl	70-400 mg/dl
Immunoglobulin M (IgM)	70 mg/dl	40-230 mg/dl
Lactate Dehydrogenase (LDH)	381 units/liter	105-233 units/liter

**Figure 1:**
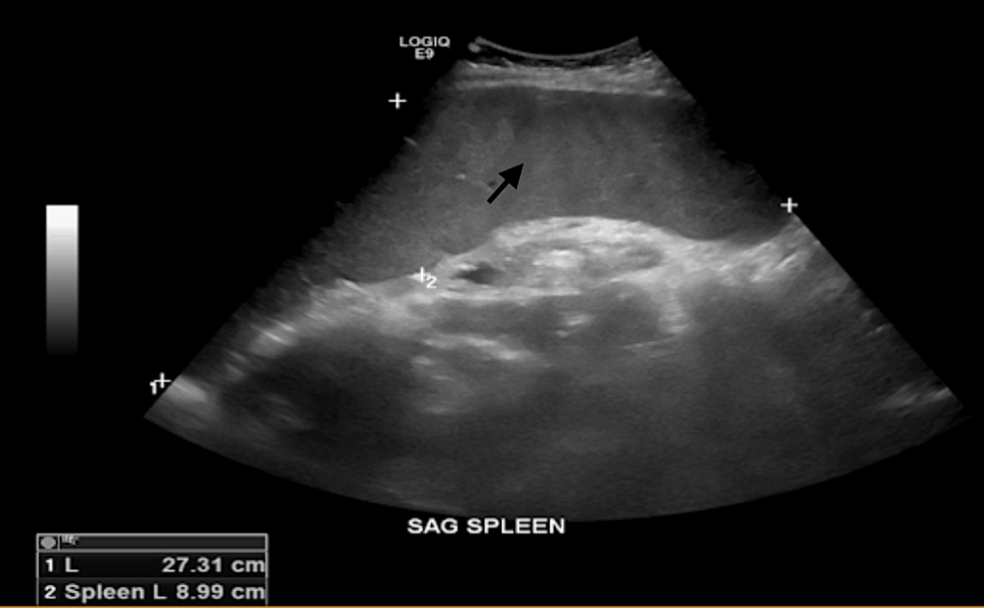
Abdominal USG showing massive splenomegaly (marked by a black arrow)

Peripheral blood smear showed mature leukocytes without dyspoiesis, some mildly enlarged lymphocytes without abnormal morphology, and rare granular lymphocytes, red cells showed moderate anisocytosis with some elliptocytes and rare teardrops; no schistocytes or spherocytes were identified. Platelets were not clumped. Bone marrow biopsy showed normocellular marrow with a small abnormal B-cell population (5-10% of leukocytes), flow cytometry detects an abnormal B-cell population with kappa positive, negative for CD5, CD10, and CD11c. BCR-ABL translocation was negative. Abdomen ultrasound (US) showed massive splenomegaly and possible hemangiomas in the right liver, two masses, or lymphadenopathy at the head of the pancreas largest diameter measuring up to 4.5 cm (Figure 1).

**Figure 2:**
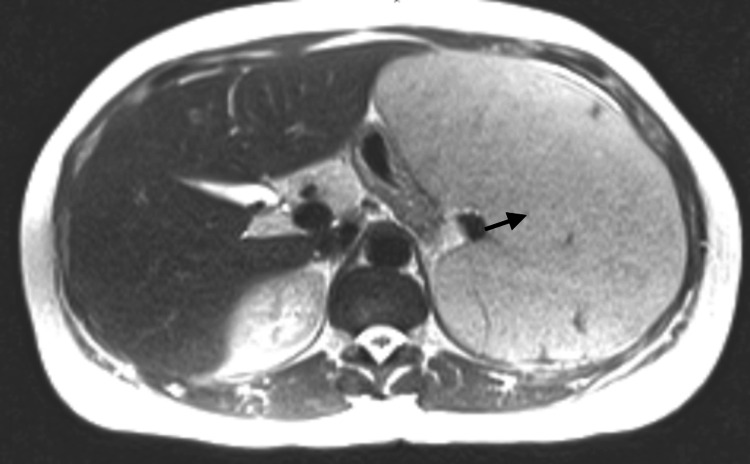
MRI abdomen with contrast showing massive splenomegaly (marked by a black arrow)

Magnetic resonance imaging (MRI) of the abdomen with contrast showed a massive spleen, 26 centimeters (cm) in circumference, 10 cm in transverse, and 17 cm in anteroposterior (AP) diameter as seen in Figure 2, hepatic hemangioma, and small hepatic cysts.

**Figure 3:**
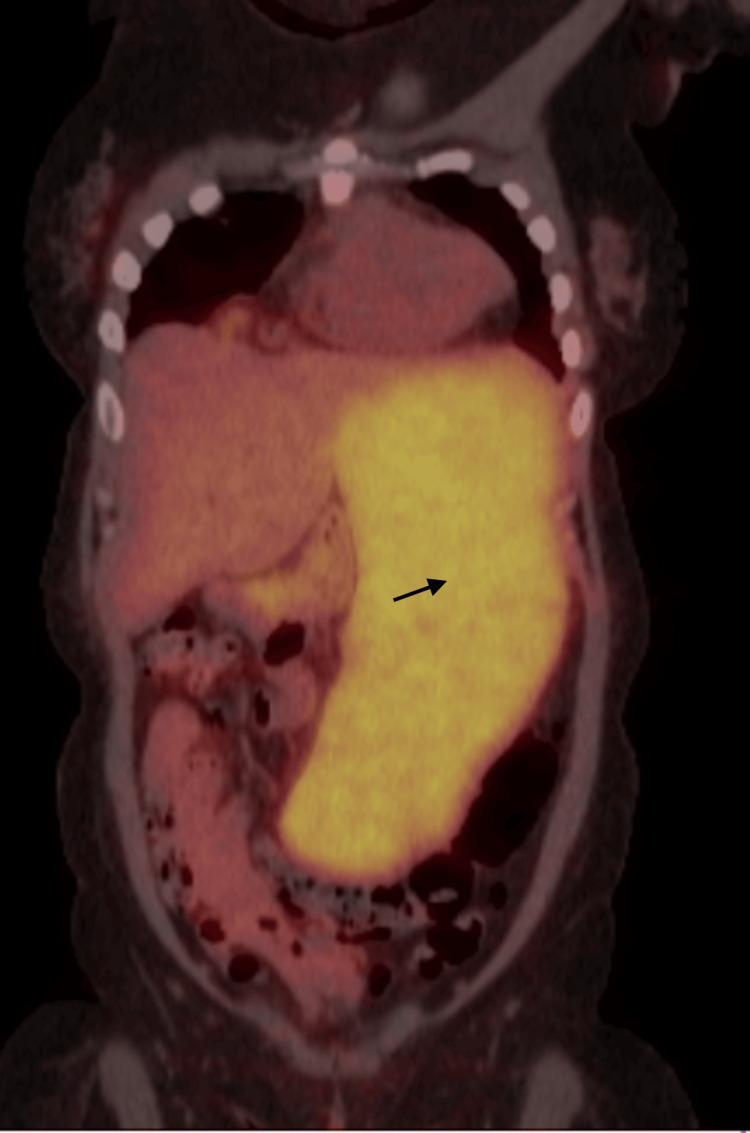
PET scan showing diffusely increased metabolic activity in the spleen with massive splenomegaly (marked by a black arrow)

The positron emission tomography (PET) scan initially showed diffusely increased metabolic activity in the spleen (maximum standardized uptake value - SUV max of 7.5) with massive splenomegaly up to 25 cm cranio-caudal as shown in Figure 3, metabolically active enlarged lymph nodes at the porta hepatis and surrounding the pancreatic head, largest measuring approximately 2.7 by 2.6 cm with SUV max of 25.6.

Given bone marrow findings, pancytopenia, and massive splenomegaly with a PET scan showing increased metabolic activity in the spleen, the patient's overall symptoms were suggestive of SMZL. Per the National Comprehensive Cancer Network guidelines, the patient was started on rituximab, standard weekly doses for a total of four doses. Given the significant burden of diseases, the patient was given allopurinol 300 mg once a day for one month to prevent tumor lysis syndrome. Before treatment with rituximab and after treatment, the patient's complete blood count is shown in Table 3.

**Table 3:** WBC, hemoglobin, and platelet levels before, after one week, after three months, and after one year of completing treatment Units of WBC: thousand/microliter (K/µL), hemoglobin: gram/deciliter (g/dl), and platelet: thousand/microliter (K/µL).

	Before treatment	After one week of completion of treatment	After three months of completion of treatment	1+ year after completing treatment
WBC (K/µL)	2.7	2.9	4.2	6.8
Hemoglobin (g/dl)	7.8	11.0	11.8	11.8
Platelets (K/µL)	77	114	147	150

**Figure 4:**
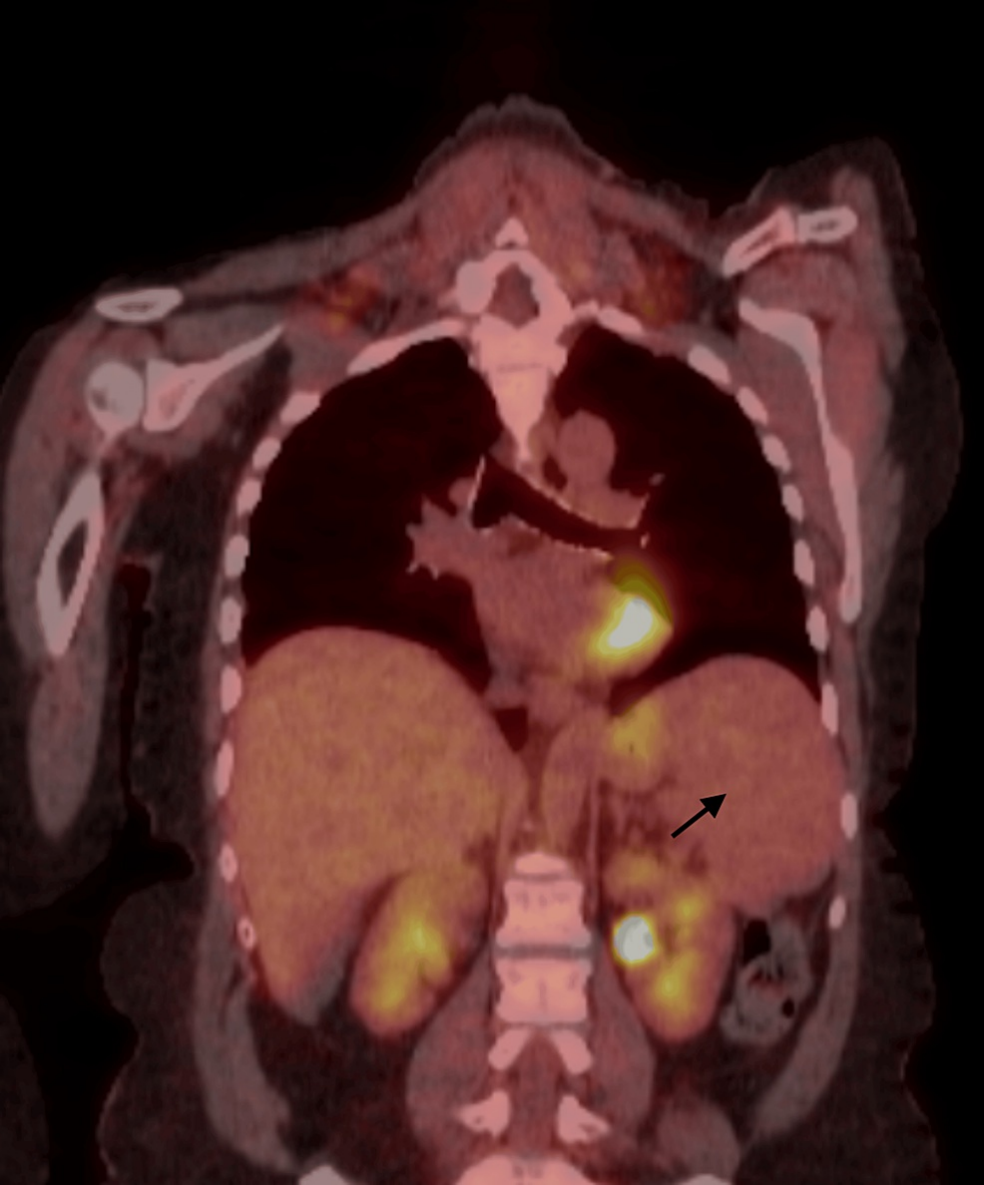
PET scan after completion of treatment showing the decrement in the size of the spleen (shown by a black arrow) and resolution of abnormal uptake seen in the prior scan.

After four weeks of completion of chemotherapy, a follow-up PET scan was done which showed interval resolution of abnormal uptake in the spleen with the decrement of the spleen size to 12.6 cm (initially up to 25 cm) and interval resolution of previously seen nodes in the abdomen, representing the complete response to the treatment as shown in Figure 4.

The patient follows regular outpatient office visits and is in remission for SMZL.

## Discussion

We present a rare case of a low-grade subtype of NHL which has a paucity of literature on clinical characteristics, diagnostic approach, and management strategy. Our patient had previously been diagnosed with APS and was later diagnosed with SMZL. In a previously reported case series of 81 patients of SMZL done by Thieblemont et al., autoimmune phenomena were reported in 16 patients including two cases of coagulation disorders, 10 cases of hemolytic anemia, and four cases of immune thrombocytopenia [9]. 

Association of SMZL as one of the malignancies among patients with positive APLAs was previously reported [7]. Furthermore, in previously reported cases, patients initially diagnosed with SMZL were found to have APLAs [4,6]. Grabska et al. suggested that the finding of autoimmune phenomena in marginal zone lymphomas (MZL) could be either secondary due to the malignancy or as a primary condition that favored the development of malignancy [10]. SMZL was never suspected in our patient with a long history of APS as there was low suspicion given her normal hematology panel in outpatient laboratory results and the patient had no manifestations of lymphoma including low-grade fever, weight loss, night sweats, and lymphadenopathy.

In previously reported cases of SMZL, suspicion of APLAs occurred after the patient was found to have thrombocytopenia, autoimmune hemolytic anemia, or pulmonary embolism [6,11]. Lupus anticoagulant (LA) was the most common antiphospholipid antibody detected which was present in up to 13% of all SMZL patients followed by anti-cardiolipin antibody (aCL) [5,6,11]. 

Previously, splenectomy was considered as the major therapy but as it is a major surgery with its own complications and as it does not cure extra splenic disease, rituximab therapy has superseded it. Splenectomy is reserved for cases refractory to rituximab therapy [12,13]. Grabska et al. suggested caution in the use of rituximab due to its association with autoimmune neutropenia and thrombocytopenia and nucleoside analogs due to their association with autoimmune hemolytic anemia [10]. However, despite the concern regarding these drugs with autoimmune phenomena, multiple studies have found resolution of APLAs after treatment with rituximab with or without chemotherapy [7,14,15]. In a study by Gebhart et al., among nine patients of SMZL with positive LA, splenectomy was not associated with persistent complete remission of LA but treatment with rituximab-bendamustine chemotherapy was [5]. A case report by Liu et al. on a patient with MZL with APLAs supported the use of bendamustine-rituximab as a reasonable first-line therapy [16]. Furthermore, SMZL patients with positive LA were suggested against splenectomy due to increased risk for splenic venous thrombosis [5]. 

Rituximab is the first-line treatment for SMZL with chemotherapy being considered as an addition to rituximab for cases with disseminated disease, constitutional symptoms, and/or signs of high-grade transformation [17,18]. 

Our patient was managed with rituximab monotherapy. The patient follows regular outpatient office visits and is in remission for SMZL.

## Conclusions

Our case report highlights the importance of a workup for possible association of malignancy in patients who are already diagnosed with APS and who present with findings of cytopenias and splenomegaly. Acute ischemic events including splenic infarction in APS could be secondary to the subtherapeutic coagulation profile. However, patients might have an underlying occult malignancy including SMZL. A high index of suspicion for SMZL should be made in patients with APS presenting with ischemic events who are found to have cytopenias and splenomegaly. Our patient had improvement with rituximab therapy. This also supports the promising advantage of medical management rather than splenectomy which has numerous complications.
